# Analysis of prognosis and treatment decisions for patients with second primary lung cancer following esophageal cancer

**DOI:** 10.3389/fonc.2022.777934

**Published:** 2022-08-16

**Authors:** Jin-luan Li, Hui Li, Qian Wu, Han Zhou, Yi Li, Yong-heng Li, Jiancheng Li

**Affiliations:** ^1^ Department of Radiation Oncology, Clinical Oncology School of Fujian Medical University, Fujian Cancer Hospital, Fuzhou, China; ^2^ Department of Radiation Oncology, Key Laboratory of Carcinogenesis Translational Research (Ministry of Education/Beijing), Peking University Cancer Hospital & Institution, Beijing, China

**Keywords:** esophageal cancer, second primary lung cancer, nomogram, competing risk analysis, prognosis, treatment decision

## Abstract

**Introduction:**

As the long-term prognosis of esophageal cancer (EC) is improving, concerns of a second primary malignancy (SPM) have increased. However, research on lung cancer as the SPM after EC is limited. Therefore, we aimed to explore the prognostic factors and clinical treatment decisions of patients with second primary lung cancer following esophageal cancer (SPLC-EC).

**Materials and methods:**

We identified the data of 715 patients with SPLC-EC from the Surveillance, Epidemiology, and End Results (SEER) database during 1975 to 2016. We established a nomogram through Cox regression modelling to predict the prognosis of patients with SPLC-EC. We determined the association between factors and cancer-specific mortality using the Fine-Gray competing risk model. Then, we performed survival analysis to evaluate the benefits of different treatment methods for overall survival (OS).

**Results:**

The multivariate analysis indicated that sex, insurance recode, age, surgery and chemotherapy 0for first primary malignancy (FPM), primary site, stage, and surgery for SPM were independent prognostic factors for OS. Using concordance indices for OS, the nomogram of our cohort showed a higher value than the SEER historic-stage nomogram (0.8805 versus 0.7370). The Fine-Gray competing risk model indicated that surgery for FPM and SPM was the independent prognostic factor for EC-specific mortality (P=0.016, hazard ratio [HR] = 0.532) and LC-specific mortality (p=0.016, HR=0.457), respectively (p<0.001). Compared to the patient group having distant metastasis, patients with localized and regional metastasis benefitted from undergoing surgery for SPM (P<0.001, P<0.001, respectively). For patients without surgery for SPM, radiotherapy (P<0.001) and chemotherapy (P<0.001) could improve OS.

**Conclusions:**

Surgery remains the mainstay for managing SPLC-EC, especially for localized and regional tumors. However, chemotherapy and radiotherapy are recommended for patients who cannot undergo surgery. These findings can have implications in the treatment decision-making for patients with SPLC-EC.

## Introduction

With the advancements in medicine, the survival of patients with malignant tumors has improved in the United States (1, 2). The incidence rate of second primary malignancy (SPM) of all cancers is 18% (3, 4). Previous studies reported that approximately one in 12 patients with cancer develop an SPM—lung cancer being the most common (5–7).

Clinical decision-making regarding the treatment of two adjacent, primary malignancies is complex. The long-term survival of patients with esophageal cancer (EC) has improved; consequently, research regarding EC complicated with an SPM is increasing (8–11). Lung cancer develops most commonly as an SPM after EC; however, only a few studies have documented second primary lung cancer (SPLC) following esophageal cancer (SPLC-EC) (4–7, 12). Although the mortality of patients with SPM is >50%, SPMs are not recommended to be treated conservatively solely based on the malignancy history (5, 13). Therefore, studies analyzing prognostic factors and treatment decisions for SPMs are crucial.

Nomograms of the Cox regression model and Fine–Gray competing risk model of SPLC-EC patients were established to forecast the prognosis and to analyze the relationship between treatment and cancer-specific mortality *via* the Surveillance, Epidemiology, and End Results (SEER) database (7, 14–16). Our research aims to provide evidence regarding prognostic factors and treatment decisions for patients with SPLC-EC.

## Patients and methods

### Patients

We conducted a retrospective review of patients with SPLC-EC based on the publicly available SEER Program (www.seer.cancer.gov) SEER*Stat Database which collected information regarding to the demographics, characteristics, and follow-up of the American approximately 30% cancer patients. Data from the database “Incidence-SEER 18 Regs Custom Date (with additional treatment fields), Nov 2018 Sub (1975–2016 varying)” was extracted and screened for SPLC-EC cases. Patients with an unknown age at diagnosis and primary malignancies other than esophageal and lung cancers were excluded. We have acknowledged and signed the SEER data use agreement (ID: 19628-Nov2019). The study did not require approval of the ethics review board.

### Statistical analysis and variables

We analyzed the enumeration and measurement data *via* SPSS V.26.0 (IBM Corp., Armonk, NY, USA). Statistical significance was set at P<0.05. The primary endpoint was overall survival (OS). We used the OS data and follow-up status of SPLC. We used univariate and multivariate analyses to establish the Cox proportional hazards regression model to estimate the potential predictors related to OS. Variables in this study are sex (female and male), race (black, white, and others), insurance recode (additional commercial health insurance, only basic health insurance or unknown), and characteristics for FPM and SPM (age, primary site, pathological type, grade, SEER historic stage, surgery, radiation, and chemotherapy).

### Nomogram model establishment, calibration, and external validation

A nomogram was drawn using the results of multivariate analysis, which uses the “rms” software package in R software V.4.0.3 (The R Foundation for Statistical Computing, Vienna, Austria) to integrate all independent prognostic factors and predicted 6-month, 1-year, and 3-year OS rates. The study uses Harrell’s C-index to assess the predictive power of the nomogram. The accuracy of this rule was verified through 1000 iterations of bootstrap resampling. The accuracy of the nomogram was established by net reclassification index calculations. In addition, the area under the receiver operating characteristic curve (AUC) was used to estimate survival predictions for 6-months, 1-year, and 3-years.

### Competing risk analysis

We set ending events and competitive events and used competitive risk models to estimate the potential predictors of these events. This model uses the “cmprsk” R software package to calculate independent prognostic factors when the outcome event and competitive event occur and draws a curve between the prognostic factors and the incidence of different events.

### Subgroup analysis of treatment

We used Kaplan–Meier curves to compare the OS between patients undergoing surgery for SPM and first primary malignancy (FPM) and SEER historic stage for SPM. This study verified the conclusions of two subgroup analyses *via* log-rank tests. Further, subgroup survival analyses were performed by Cox regression modeling of the data from patients undergoing radiotherapy and chemotherapy.

## Results

### Patient characteristics

We identified 3426 patients with second primary esophageal cancer diagnosed between 1975 and 2016 from the SEER database. Of them, the number of cases of lung cancer (SPLC) was the largest among SPMs (715 patients [20.87%]). The remaining SPMs included 427 patients (12.46%) with mouth, nose, and throat cancer; 401 patients (11.70%) with prostate cancer; 271 patients (7.91%) with gastric cancer; and 105 patients (3.06%) with breast cancer.

This study included data of 715 SPLC-EC patients, including 533 men (74.5%) and 182 women (25.5%). The characteristics of SPLC-EC patients are summarized in **Table 1**. The median ages at diagnosis of FPM and SPM were 66 and 69 years, respectively. SPMs were localized in 229 patients (32.0%), regional in 134 patients (18.7%), distant in 195 patients (27.3%), and unknown in 157 patients (22.0%). Surgery for SPM was performed in 191 patients (26.7%); 524 patients (73.3%) did not undergo surgery. Radiotherapy for SPM was performed in 271 patients (37.9%) and was not performed in 444 patients (62.1%); 252 patients (35.2%) underwent chemotherapy for SPM and 463 patients (64.8%) did not.

**Table 1:** Characteristics and Variables of SPLC-EC Patients Associated with OS According to the Cox Proportional Hazards Regression Model.

Characteristics	Descriptive analysis			Univariable analysis					Multivariable analysis			
n	%		P value^a^	HR	95%CI			P value^a^	HR	95%CI	
Ending Event												
Alive	88	12.3										
Esophagus Cancer-specific Mortality	255	35.7										
Lung Cancer-specific Mortality	254	35.5										
Others	118	16.5										
Gender												
Female	182	25.5		Reference					Reference			
Male	533	74.5		0.029	1.229	1.022	1.478		0.045	1.212	1.004	1.463
Race				0.055					0.444			
Black	119	16.6		Reference					Reference			
White	564	78.9		0.187	0.868	0.703	1.071		0.322	0.889	0.704	1.122
Others	32	4.5		0.207	1.304	0.863	1.971		0.755	1.071	0.695	1.650
Insurance Recode												
Insured	267	37.3		Reference					Reference			
No/Unknown	448	62.7		<0.001	1.442	1.216	1.710		0.008	1.273	1.065	1.522
Age for FPM(years)												
<65	317	44.3		Reference					Reference			
≥65	398	55.7		0.004	1.260	1.075	1.477		0.005	1.261	1.071	1.485
Primary Site for FPM				0.078					0.262			
Upper third of esophagus	53	7.4		Reference					Reference			
Middle third of esophagus	144	20.1		0.644	0.925	0.664	1.288		0.933	0.985	0.694	1.398
Lower third of esophagus	364	50.9		0.126	0.788	0.581	1.069		0.184	0.792	0.561	1.117
Others	98	13.7		0.778	1.052	0.740	1.496		0.867	1.031	0.720	1.476
NOS	56	7.8		0.983	0.996	0.669	1.483		0.388	0.829	0.542	1.269
Pathological Type(ICD-O-3) for FPM				0.068					0.839			
Squamous Carcinoma	370	51.7		Reference					Reference			
Adenocarcinoma	292	40.8		0.034	0.836	0.709	0.986		0.895	0.985	0.788	1.232
Others	53	7.4		0.702	1.061	0.785	1.432		0.611	1.086	0.790	1.495
Grade for FPM				0.413								
Well	44	6.2		Reference								
Moderate	271	37.9		0.072	0.732	0.521	1.028					
Poor	227	31.7		0.101	0.750	0.532	1.058					
Undifferentiated	12	1.7		0.837	0.934	0.489	1.786					
Unknown	161	22.5		0.093	0.737	0.515	1.052					
SEER Historic Stage for FPM				0.207								
Distant	101	14.1		Reference								
Localized	274	38.3		0.182	0.848	0.665	1.081					
Regional	239	33.4		0.048	0.779	0.607	0.998					
Unknown	101	14.1		0.633	0.929	0.688	1.255					
Surgery for FPM												
Not Performed	430	60.1		Reference					Reference			
Performed	285	39.9		<0.001	0.679	0.576	0.799		0.009	0.782	0.650	0.941
Radiation for FPM												
Not Performed	238	33.3		Reference								
Performed	477	66.7		0.565	0.952	0.806	1.125					
Chemotherapy for FPM												
No/Unknown	282	39.4		Reference					Reference			
Performed	433	60.6		<0.001	0.743	0.634	0.872		0.007	0.786	0.660	0.936
Age for SPM(years)												
<65	235	32.9		Reference					Reference			
≥65	480	67.1		0.047	1.185	1.002	1.402		0.940	1.011	0.759	1.348
Primary Site for SPM				<0.001					<0.001			
Main Bronchus	40	5.6		Reference					Reference			
Upper Lobe	347	48.5		<0.001	0.465	0.330	0.653		<0.001	0.498	0.350	0.707
Middle Lobe	33	4.6		0.300	0.774	0.477	1.256		0.675	0.899	0.547	1.479
Lower Lobe	194	27.1		<0.001	0.524	0.368	0.747		0.006	0.598	0.415	0.863
NOS	101	14.1		0.821	1.044	0.717	1.522		0.266	0.801	0.542	1.183
Pathological Type (ICD-O-3) for SPM				<0.001					0.056			
Squamous Carcinoma	239	33.4		Reference					Reference			
Adenocarcinoma	223	31.2		<0.001	0.688	0.563	0.840		0.029	0.789	0.638	0.976
Small Cell Carcinoma	60	8.4		0.250	1.188	0.886	1.593		0.334	1.182	0.842	1.659
Others	193	27		0.777	1.029	0.843	1.256		0.940	0.991	0.789	1.246
Grade for SPM				0.012					0.023			
Well	52	7.3		Reference					Reference			
Moderate	135	18.9		0.358	1.181	0.828	1.685		0.379	1.183	0.814	1.719
Poor	168	23.5		0.008	1.582	1.124	2.226		0.471	1.142	0.796	1.638
Undifferentiated	32	4.5		0.027	1.707	1.063	2.742		0.418	1.237	0.739	2.070
Unknown	328	45.9		0.012	1.516	1.095	2.098		0.341	0.844	0.594	1.197
SEER Historic Stage for SPM				<0.001					<0.001			
Distant	195	27.3		Reference					Reference			
Localized	229	32		<0.001	0.334	0.271	0.412		<0.001	0.392	0.310	0.495
Regional	134	18.7		<0.001	0.441	0.348	0.558		<0.001	0.568	0.441	0.733
Unknown	157	22		0.002	0.706	0.564	0.883		<0.001	0.641	0.506	0.813
Surgery for SPM												
Not Performed	524	73.3		Reference					Reference			
Performed	191	26.7		<0.001	0.441	0.365	0.534		<0.001	0.444	0.345	0.572
Radiation for SPM												
Not Performed	444	62.1		Reference					Reference			
Performed	271	37.9		0.443	0.938	0.797	1.104		<0.001	0.706	0.590	0.845
Chemotherapy for SPM												
No/Unknown	463	64.8		Reference					Reference			
Performed	252	35.2		0.271	0.912	0.773	1.075		<0.001	0.699	0.579	0.844

SPLC-EC, second primary lung cancer in esophagus cancer; OS, overall survival; FPM, first primary malignancy; SPM, second primary malignancy; HR, hazard ratio; SEER, Surveillance, Epidemiology, and End Results.

^a^Bolded values are statistically significant.

### Cox regression model and nomogram

Multivariate analysis indicated that sex (P=0.045, hazard ratio [HR]=1.212, 95% confidence interval [CI] 1.004–1.463), insurance recode (P=0.008, HR=1.273, 95% CI 1.065–1.522), age at FPM diagnosis (P=0.005, HR=1.261, 95% CI 1.071–1.485), surgery for FPM (P=0.009, HR=0.782, 95% CI 0.650–0.941), chemotherapy for FPM (P=0.007, HR=0.786, 95% CI 0.660–0.936), primary site of SPM (P<0.001; main bronchus vs. upper lobe, P<0.001, HR=0.498, 95% CI 0.350–0.707; main bronchus vs. lower lobe, P=0.006, HR=0.598, 95% CI 0.415–0.863), SEER historic stage of SPM (P<0.001; distant stage vs. localized stage, P<0.001, HR=0.392, 95% CI 0.310–0.495; distant stage vs. regional stage, P<0.001, HR=0.568, 95% CI 0.441–0.733), and surgery for SPM (P<0.001, HR=0.444, 95% CI 0.345–0.572) were the independent prognostic factors for OS.

**Figure 1:**
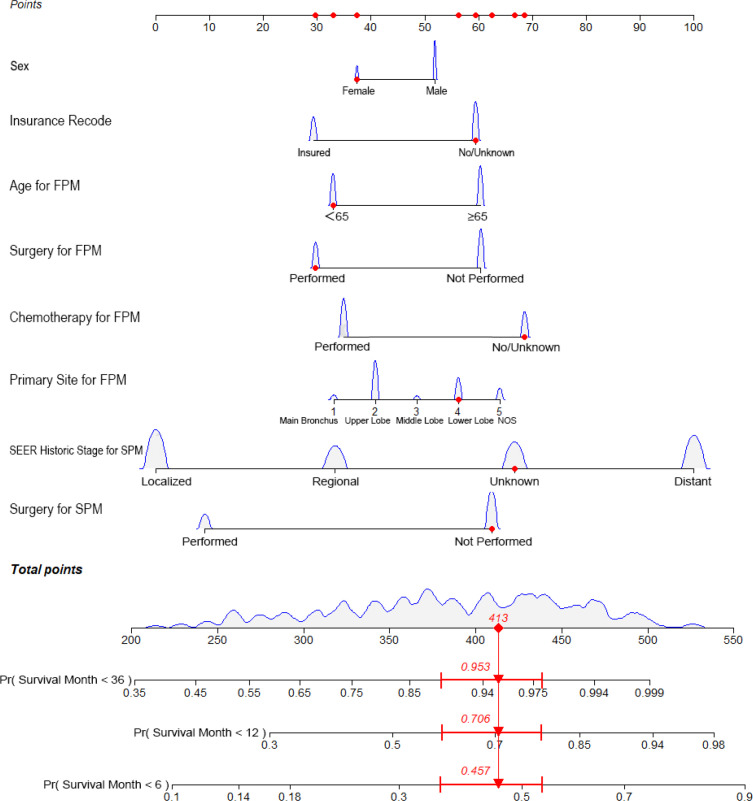
Nomogram predicting the 6-month, 1-year, and 3-year OS rates of patients with second primary lung cancer in esophagus cancer. The nomogram summed the points identified on the scale for each variable. FPM, first primary malignancy; SPM, second primary malignancy; OS, overall survival.

**Figure 2:**
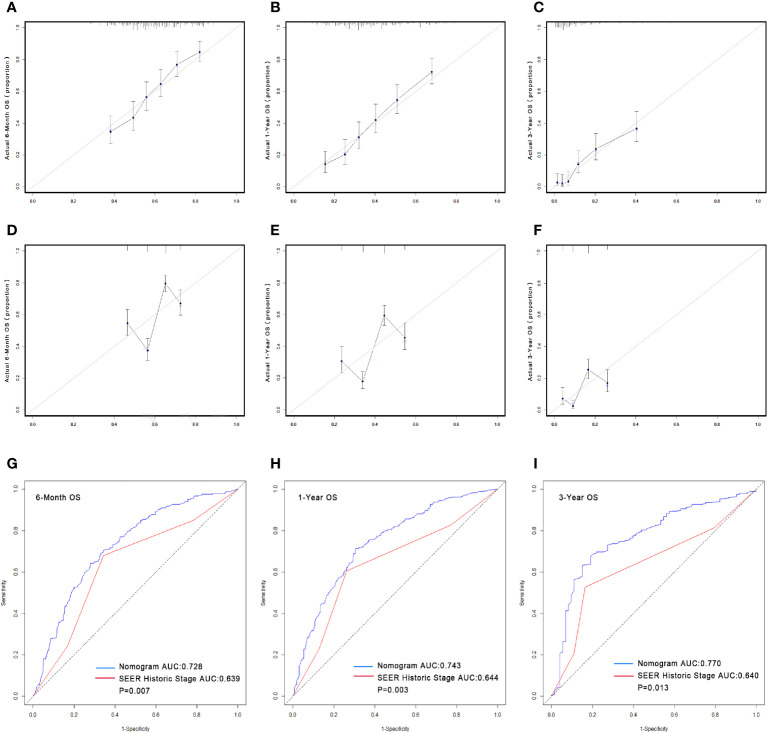
Calibrations of the nomograms and stage for predicting survival rates **(A–F)**. The x-axis represents the nomogram-predicted survival rates, whereas the y-axis represents the actual survival rates. All predictions lie within a 10% margin of error (within the dashed lines). **(A)** Calibration of the nomogram for predicting the 6-month OS rate. **(B)** Calibration of the nomogram for predicting the 1-year OS rate. **(C)** Calibration of the nomogram for predicting the 3-year OS rate. **(D)** Calibration of the stage for predicting the 6-month OS rate. **(E)** Calibration of the stage for predicting the 1-year OS rate. **(F)** Calibration of the stage for predicting the 3-year OS rate. Comparison of the AUCs of the nomogram and SEER historic stage for predicting survival rates. The blue lines represent nomogram predicted survival rates, whereas the red lines represent SEER historic stage predicted survival rates. AUCs of the two models predict OS rates at 6 months **(G)**, 1 year **(H)** and 3 years **(I)**. OS, overall survival; AUC, area under the curve; SEER, Surveillance, Epidemiology, and End Results.

The predictive nomogram was plotted for OS rates at 6 -months, 1-year, and 3-years considering the above-mentioned factors (**Figure 1**). The C-index for OS was 0.8805 (95% CI 0.8473–0.9138), which was higher than that for SEER historic stage (0.7370, 95% CI 0.6877–0.7862). The calibration curves of the nomogram model and the SEER historic-stage model were plotted (**Figures 2A–F**). Compared with the SEER historic-stage model, the calibration curves of the nomogram demonstrated a higher accuracy in classification capability for predicting OS rates at 6-months, 1-year, and 3-years. Further, compared with the SEER historic stage, reclassification accuracy of the nomogram model for OS rates at 6-months, 1-year, and 3-years increased by 52.053% (95% CI 36.525%–69.486%), 56.397% (95% CI 40.794%–74.501%), and 72.111% (95% CI 48.774%–99.999%), respectively. Furthermore, the AUCs were plotted to assess the predictive power of the nomogram and the SEER historic-stage models (**Figures 2G–I**). The AUCs for OS rates indicated that the predictive ability of the nomogram we constructed was significantly stronger than that of the SEER historic stage (6-month OS: nomogram AUC=0.728 vs. SEER historic stage AUC=0.639, P=0.007; 1-year OS: nomogram AUC=0.743 vs. SEER historic stage AUC=0.644, P=0.003; 3-year OS: nomogram AUC=0.770 vs. SEER historic stage AUC=0.640, P=0.013; respectively).

### Ending event-based regulation and competing risk model

**Figure 3:**
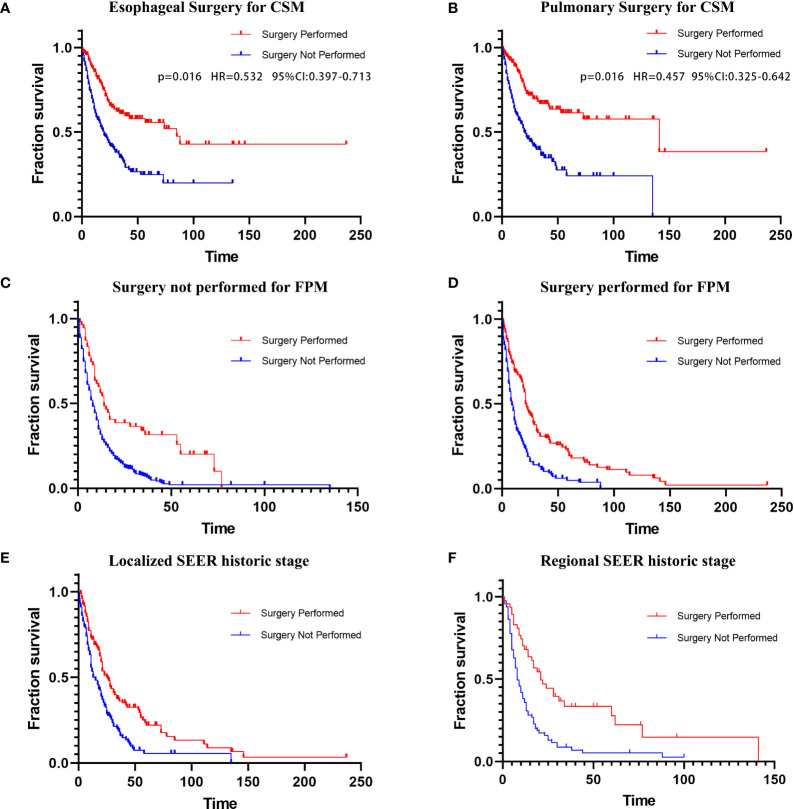
**(A)** Esophageal CSM in patients undergoing esophageal surgery; **(B)** Lung CSM in patients undergoing pulmonary surgery. Kaplan–Meier curves of overall survival in patients undergoing surgery for SPM; **(C)** Surgery not performed for FPM; **(D)** Surgery performed for FPM; **(E)** Localized SEER historic stage; **(F)** Regional SEER historic stage. CSM, cancer-specific mortality; FPM, first primary malignancy; SPM, second primary malignancy; SEER, Surveillance, Epidemiology, and End Results.

In the ending event-based analysis, 254 patients (35.5%) died from SPLC, 255 patients (35.7%) died from FPM, and 206 patients (28.8%) had other endpoint events (**Table 1**). Therefore, we established a Fine–Gray competing risk model to analyze prognostic factors related to specific causes of death in patients with SPLC-EC (**Figures 3A, B**). We set lung cancer-specific mortality as the final event and esophageal cancer-specific mortality as the competitive event. The Fine–Gray proportional sub-distribution risk model indicated that surgery for SPM was an independent risk determinant for lung cancer-specific mortality in SPLC-EC patients (P=0.016, HR=0.457, 95% CI 0.325–0.642). Surgery for FPM was an independent risk factor for esophageal cancer-specific mortality (P=0.016, HR=0.532, 95% CI 0.397–0.713).

### Survival benefits of differential treatment after SPLC diagnosis

A subgroup analysis of surgical treatment status found that after FPM diagnosis, 285 patients (39.86%) underwent cancer-directed surgery, while 430 patients (60.14%) did not undergo surgery (**Table 2**). Among those who underwent surgical treatment, 135 patients (47.4%) underwent re-operation after SPM diagnosis (group 1; average survival months: 38.510; median survival months: 21; 95% CI 18.233–23.767). The average and the median survival times of the 150 SPM patients (52.6%) who did not undergo re-operation (group 2) were 14.439 and 8 months, respectively (95% CI 5.871–10.129). Among patients who did not undergo surgery after an FPM diagnosis, 56 patients (13%) underwent surgery after an SPM diagnosis (group 3; average survival months: 28.777; median survival months: 14; 95% CI 9.391–18.609). The average and the median survival of the 374 patients (87%) who did not undergo surgery for either FPM or SPM (group 4) was 12.830 months and 7 months, respectively (95% CI 5.716–8.284). The Kaplan–Meier curves and log-rank tests were applied to compare the four groups (group 1 versus group 2: P<0.001; group 3 vs. group 4: P<0.001; respectively) in survival (**Figures 3C, D**).

**Table 2:** Surgery for SPM Associated with Overall Survival for SPLC-EC Patients in Surgery for FPM and SEER Historic Stage for SPM of Subgroup According to the Kaplan-Meier Subsistence Analysis.

		Descriptive analysis			Mean survival time					Median survival time					Long-rank test
		n	%		mean value	standard deviation	95%CI			median value	standard deviation	95%CI			p value^a^
**Surgery for FPM: Not Performed**		430			15.510	1.371	12.823	18.197		8.000	0.581	6.862	9.138		<0.001
Surgery for SPM															
Not Performed		374	87		12.830	1.178	10.521	15.139		7.000	0.655	5.716	8.284		
Performed		56	13		28.777	3.866	21.199	36.355		14.000	2.351	9.391	18.609		
**Surgery for FPM: Performed**	** **	285			26.520	2.694	21.239	31.801		12.000	1.731	8.608	15.392		<0.001
Surgery for SPM															
Not Performed		150	52.6		14.439	1.668	11.170	17.708		8.000	1.086	5.871	10.129		
Performed		135	47.4		38.510	4.724	29.251	47.769		21.000	1.412	18.233	23.767		
**SEER Historic Stage for SPM: Localized**	** **	229			32.881	3.512	25.998	39.764		19.000	1.584	15.896	22.104		<0.001
Surgery for SPM															
Not Performed		130	56.8		22.945	2.978	17.109	28.782		13.000	1.861	9.352	16.648		
Performed		99	43.2		44.437	6.151	32.382	56.492		25.000	2.861	19.392	30.608		
**SEER Historic Stage for SPM: Regional**	** **	134			25.288	3.652	18.129	32.446		11.000	1.372	8.310	13.690		<0.001
Surgery for SPM															
Not Performed		87	64.9		14.379	2.280	9.911	18.847		21.000	4.283	12.606	29.394		
Performed		47	35.1		43.440	8.450	26.878	60.003		11.000	1.372	8.310	13.690		
**SEER Historic Stage for SPM: Distant**		195			7.739	0.711	6.345	9.133		5.000	0.462	4.094	5.906		0.271
Surgery for SPM															
Not Performed		179	91.8		7.493	0.725	6.072	8.913		4.000	0.515	2.991	5.009		
Performed		16	8.2		10.438	3.052	4.455	16.420		6.000	2.000	2.080	9.920		

SPM, second primary malignancy; SPLC-EC, second primary lung cancer in esophagus cancer; FPM, first primary malignancy; SEER, Surveillance, Epidemiology, and End Results.

^a^Bolded values are statistically significant.

Another subgroup analysis of surgery for SPM also used the Kaplan–Meier method. We divided the cohort into distant, localized, and regional groups based on the SEER historic stage (**Table 2**). In the localized group, both the average and median survival times of patients who underwent surgery for SPM were significantly longer than those of patients who did not (average: 44.437 versus 22.945 months; median: 25 vs 13 months; log-rank test: P<0.001). In the regional group, the average survival time of patients who underwent surgery for SPM was significantly longer than that of patients who did not (43.44 vs 14.379 months); however, the median survival time was longer in patients who did not undergo surgery (21 versus 11 months; log-rank test: P <0.001). The log-rank test for the distant group showed no statistical significance in survival times between patients who underwent surgery and patients who did not (P =0.271). Further, we used Kaplan–Meier curves to illustrate the impact of surgery on survival at different cancer stages (**Figures 3E, F**).

To analyze prognostic differences in radiotherapy and chemotherapy for SPM, a Cox proportional hazard regression model was applied. After SPLC diagnosis, the cohort was split into the surgery (524 patients, 73.3%) and non-surgery groups (158 patients, 26.7%). Among patients of the SPM group with surgery, radiotherapy and chemotherapy were not statistically significant in the Cox regression model (P=0.778, P=0. 944, respectively). While radiotherapy and chemotherapy for patients of the SPM group without surgery were statistically significant (P<0.001, HR=0.660, 95% CI: 0.544–0.802; P<0.001, HR=0.657, 95% CI 0.535–0.808; respectively) (**Table 3**).

**Table 3:** Radiotherapy and chemotherapy associated with OS for SPLC-EC patients in surgery for SPM according to the Cox proportional hazards regression model.

	Descriptive analysis			Univariable analysis					Multivariable analysis			
	n	%		P value^a^	HR	95%CI			P value^a^	HR	95%CI	
**Surgery for SPM: Not Performed**	524											
Radiotherapy for SPM												
Not Performed	286	54.6		Reference					Reference			
Performed	238	45.4		<0.001	0.624	0.519	0.749		<0.001	0.66	0.544	0.802
Chemotherapy for SPM												
No/Unknown	312	59.5		Reference					Reference			
Performed	212	40.5		<0.001	0.715	0.594	0.861		<0.001	0.657	0.535	0.808
**Surgery for SPM: Performed**	191											
Radiotherapy for SPM												
Not Performed	158	82.7		Reference					Reference			
Performed	33	17.3		0.306	1.244	0.819	1.89		0.778	0.926	0.541	1.585
Chemotherapy for SPM												
No/Unknown	151	79.1		Reference					Reference			
Performed	40	20.9		0.948	0.987	0.670	1.454		0.944	1.018	0.614	1.689

OS, overall survival; SPLC-EC, second primary lung cancer in esophagus cancer; SPM, second primary malignancy; HR, hazard ratio.

^a^Bolded values are statistically significant.

## Discussion

As the incidence of SPM increases, research on the monitoring of, prognosis of, and treatment decisions for SPM has become significant (2, 3, 6). This large-scale cohort study found that the independent prognostic factors for OS of patients with SPLC-EC were sex, insurance recode, age at FPM diagnosis, surgery for FPM, chemotherapy for FPM, primary site of SPM, SEER historic stage of SPM, and surgery for SPM. We first established an interactive nomogram for patients with SPLC-EC that displayed a comparatively better prognostic discrimination and predictive accuracy for OS rates than the SEER historic stage. Competing risk models suggested that surgery is the preferred treatment for patients having SPLC-EC without distant metastasis. Further, radiotherapy and chemotherapy were shown to provide survival benefits to patients who cannot undergo surgery. These results may guide clinicians in the diagnosis and treatment of patients with SPLC-EC.

Surgery is a momentous treatment for lung cancer (17–19). For first primary lung cancer, Donington et al. (20) reported that the gold standard treatment for early stage (I/II) was lobectomy. In addition, Martini et al. (21) and Naruke et al. (22) reported that patients with N2-3 had reached 5-year OS at around 30% by systematic radical mediastinal lymphadenectomy. For SPLC, Song et al. (23) reported that patients who underwent surgery obviously had improved long-term survival (P<0.001, HR=0.36, 95% CI 0.30–0.44), reporting 3-year OS rates of 66.0%. However, the role of surgery in patients with SPLC-EC remains unclear. In this study, surgery was an independent prognostic determinant of OS (P<0.001, HR=0.444, 95% CI 0.345–0.572). According to our established nomogram (**Figure 1**), surgical performance for both primary cancers had a significant effect in survival, especially for patients with SPLC. The competitive risk model also confirmed that in the two ending events, lung and EC-specific mortality, surgery was strongly negatively correlated with cancer-specific death (P=0.016, HR=0.457, 95% CI 0.325–0.642 and P=0.016, HR=0.532, 95% CI 0.397–0.713, respectively). Subgroup analysis showed that compared with non-surgical patients, surgery can prolong the survival (log-rank test, P<0.001), especially for patients with localized and regional malignancies (both P<0.001). Even if patients with SPLC-EC underwent surgery twice, the survival effect was still excellent (log-rank test, P<0.001). Thus, active surgery is associated with favorable long-term survival.

Chemotherapy and radiotherapy play significant roles in lung cancer management (24–27). Bradley et al. (28) reported that concurrent chemotherapy and radiotherapy is the standard treatment for locally advanced lung cancer. Zukin et al. (29) reported that chemotherapy is extremely important for OS of patients with advanced lung cancer (progression-free survival: HR=0.46, P<0.001; OS: HR=0.62, P=0.001). Burdett et al. (30) and Le Chevalier et al. (31) reported that neoadjuvant and adjuvant chemotherapy had improved the 5-year OS of patients with primary lung cancer by 6% and 4%, respectively. However, the efficacy of radiotherapy and chemotherapy in patients with SPLC-EC remains uncertain. Multivariate analysis in this study found that radiotherapy and chemotherapy for SPLC had statistically significant OS rates (P<0.001, HR=0.706; P<0.001, HR=0.699, respectively). Similar results about the radiotherapy and chemotherapy were found in the subgroup analysis of patients who did not undergo surgery (P<0.001, HR=0.715; P<0.001, HR=0.657; respectively). Therefore, patients with SPLC-EC may benefit from radiotherapy and chemotherapy.

Sex is considered a significant variable affecting lung cancer prognosis (32–34). Wisnivesky et al. (35) reported that among lung cancer patients, the 5-year relative survival rates of men were lower than that of women (38% vs. 46%; P<0.0001). For patients with SPLC, Song et al. (23) reported that the 3-year OS rates and multivariate analysis of OS for men were worse than those for women (30.4% vs. 42.8%, P<0.001; log-rank test, P=0.004, HR=1.28). This study also revealed sex as a prognostic determinant for SPLC-EC patients (P=0.045, HR=1.212, 95% CI 1.004–1.463).

Likewise, age is another recognized important risk contributor for lung cancer (36, 37). Owonikoko et al. (38) reported that the older the lung cancer patients, their 5-year OS rates decreased (aged ≤69 years: 15.5%, aged 70–79 years: 12.3%, aged ≥80 years: 7.4%, respectively; P<0.0001). Song et al. (23) further reported that the SPLC patients aged ≤ 64 years had better 3-year OS rates than patients aged >65 years (39.3% vs. 33.6%, P=0.024, HR=1.18). Similarly, our study also found that age at FPM diagnosis ≥65 years is a poor prognostic determinant (P=0.005, HR=1.261) for patients with SPLC-EC. This might be attributed to worse physical conditions, poor tolerance to treatment, and worsening cancer stages in older patients.

This large-scale retrospective cohort study had few limitations. First, the study spanned a long duration (1975–2016), was retrospective in nature, and had selection bias. Second, potential confounders, such as concrete methods of surgery, chemotherapy, radiotherapy, and reason for treatment selection, were unmeasured and thus not reported in the SEER database, which may have influenced the results. Lastly, cigarette-smoking data are not recorded in the SEER database, we could not study its impact on the prognosis of SPLC-EC. Future research should address the above-mentioned deficiencies, which would alleviate the conditions of patients with SPLC-EC.

## Conclusion

In our study, an interactive nomogram based on independent prognostic factors was established, and its prediction for OS was comparatively better than that of the SEER historic stage of patients with SPLC-EC. Fine–Gray competing risk models identified surgery as the preferred treatment option for patients with SPLC-EC, especially those with localized and regional malignancy. When patients with SPLC-EC cannot undergo surgery, chemotherapy and radiotherapy are strongly recommended. These findings may guide the treatment decisions for patients with SPLC-EC in the future.

## Data availability statement

The datasets presented in this study can be found in online repositories. The names of the repository/repositories and accession number(s) can be found in the article/supplementary material.

## Author contributions

Study concept: J-LL, HL, J-CL. Data analysis: HL, QW, HZ, YL. Manuscript preparation: J-LL, HL. Critical revision: J-LL, J-CL, Y-HL Study supervision: J-LL, HL. Approval of final manuscript: All authors. All authors agree to be accountable for the content of the work.

## Funding

This research was funded by the Fujian Province Natural Science Foundation, 2021J01433; National Clinical Key Specialty Construction Program, 2021; Fujian Provincial Clinical Research Center for Cancer Radiotherapy and Immunotherapy, 2020Y2012.

## Acknowledgments

The authors sincerely thank the Surveillance, Epidemiology, and End Results (SEER) program for their efforts in establishing the SEER database. We would like to thank Editage for linguistic assistance during the preparation of this manuscript.

## Conflict of interest

The authors declare that the research was conducted in the absence of any commercial or financial relationships that could be construed as a potential conflict of interest.

## Publisher’s note

All claims expressed in this article are solely those of the authors and do not necessarily represent those of their affiliated organizations, or those of the publisher, the editors and the reviewers. Any product that may be evaluated in this article, or claim that may be made by its manufacturer, is not guaranteed or endorsed by the publisher.

## Author disclaimer

The authors state that the views expressed in the submitted article are their own and not an official position statement of the institution or funder.
